# Metformin and longevity (METAL): a window of opportunity study investigating the biological effects of metformin in localised prostate cancer

**DOI:** 10.1186/s12885-017-3458-3

**Published:** 2017-07-21

**Authors:** Danielle Crawley, Ashish Chandra, Massimo Loda, Cheryl Gillett, Paul Cathcart, Ben Challacombe, Gary Cook, Declan Cahill, Aida Santa Olalla, Fidelma Cahill, Gincy George, Sarah Rudman, Mieke Van Hemelrijck

**Affiliations:** 1 Division of Cancer Studies, Cancer Epidemiology Group, Research Oncology, King’s College London, 3rd Floor, Bermondsey Wing, Guy’s Hospital, London, SE1 9RT UK; 2 Guy’s and St Thomas’ NHS Foundation Trust, London, UK; 3 Department of Medical Oncology, Dana-Farber Cancer Institute, Boston, USA; 4 Division of Cancer Studies, King’s College London, London, UK; 5 Division of Imaging Sciences and Biomedical Engineering, King’s College London, London, UK; 6 Royal Marsden NHS Foundation Trust, London, UK

## Abstract

**Background:**

Metformin is a biguanide oral hypoglycaemic agent commonly used for the treatment of type 2 diabetes mellitus. In addition to its anti-diabetic effect, metformin has also been associated with a reduced risk of cancer incidence of a number of solid tumours, including prostate cancer (PCa). However, the underlying biological mechanisms for these observations have not been fully characterised in PCa. One hypothesis is that the indirect insulin lowering effect may have an anti-neoplastic action as elevated insulin and insulin like growth factor − 1 (IGF-1) levels play a role in PCa development and progression. In addition, metformin is a potent activator of activated protein kinase (AMPK) which in turn inhibits the mammalian target of rapamycin (mTOR) and other signal transduction mechanisms. These direct effects can lead to reduced cell proliferation. Given its wide availability and tolerable side effect profile, metformin represents an attractive potential therapeutic option for men with PCa. Hence, the need for a clinical trial investigating its biological mechanisms in PCa.

**Methods:**

METAL is a randomised, placebo-controlled, double-blind, window of opportunity study investigating the biological mechanism of metformin in PCa. 100 patients with newly-diagnosed, localised PCa scheduled for radical prostatectomy will be randomised 1:1 to receive metformin (1 g b.d.) or placebo for four weeks (+/− 1 week) prior to prostatectomy. Tissue will be collected from both diagnostic biopsy and prostatectomy specimens. The primary endpoint is the difference in expression levels of markers of the Fatty acid synthase (FASN)/AMPK pathway pre and post treatment between the placebo and metformin arms. Secondary endpoints include the difference in expression levels of indicators of proliferation (ki67 and TUNEL) pre and post treatment between the placebo and metformin arms. METAL is currently open to recruitment at Guy’s and St Thomas’ Hospital and the Royal Marsden Hospital, London.

**Discussion:**

This randomised placebo-controlled double blinded trial of metformin vs. placebo in men with localised PCa due to undergo radical prostatectomy, aims to elucidate the mechanism of action of metformin in PCa cells, which should then enable further larger stratification trials to take place.

**Trial registration:**

EudraCT number 2014–005193-11. Registered on September 09, 2015.

**Electronic supplementary material:**

The online version of this article (doi:10.1186/s12885-017-3458-3) contains supplementary material, which is available to authorized users.

## Background

The incidence of prostate cancer (PCa) has significantly increased over the past decades and will remain a significant health burden in years ahead. Patients presenting with localised disease at diagnosis are categorised into low, intermediate or high risk based on clinical stage, prostate specific antigen (PSA) level and histopathological Gleason score [1]. Current treatment options for men with intermediate and high risk disease include radical prostatectomy (open, laparoscopic or robotic) and radiotherapy with Neoadjuvant/adjuvant hormone therapy [2]. However, due to the risk of relapse in these groups, Neoadjuvant treatment has been investigated, but with disappointing results [3].

Type 2 Diabetes (T2DM) or impaired glucose tolerance are included in the cluster of disorders which comprise the metabolic syndrome (MetS) [4]. During the last decade, studies have investigated whether MetS is involved in the aetiology of PCa [5–7] [8, 9]. A meta-analysis to quantify the risk of PCa related to MetS found a pooled relative risk of 1.54 (95% CI:1.23–1.94) [4]. Recent studies have also suggested that the presence of MetS or some of its features is associated with higher grade disease in men with PCa and can lead to more rapid progression to castrate resistant PCa [10, 11].

Metformin (1,1-dimethylbiguanide hydrochloride) is a biguanide class of oral hypoglycaemic agent and commonly used for the treatment of T2DM. Metformin inhibits gluconeogenesis and reduces circulating levels of insulin [12]. It is also thought to play a role in lowering triglycerides and LDL cholesterol levels [13]. The usual dose is 2 g daily in divided doses and mild gastrointestinal discomfort with diarrhoea is the most common side effect (>10%). Other common side effects include: nausea, vomiting and abdominal pain. However, if dose escalation is perfomed carefully most patients are able to receive maximum drug dosing. Lactic acidosis is a very rare, but a serious adverse event [14]. To limit the risk of lactic acidosis, patients with risk factors for its development will be excluded from the study (renal impairment, hypoxia, congestive heart failure).

In addition to the anti-diabetic effect, metformin has also been associated with a reduced risk of various cancers, including PCa incidence and mortality in epidemiological studies [15–17]. However, the underlying biological mechanisms for these observations have yet to be fully characterised [18]. One hypothesis is that indirect insulin lowering effect may have an anti-neoplastic effect as elevated insulin and insulin like growth factor − 1 (IGF-1) levels play a role in prostate cancer development and progression [19]. In addition, metformin is also a potent activator of activated protein kinase (AMPK), which in turn inhibits the mammalian target of rapamycin (mTOR) and other protein synthesis. These direct effects can lead to reduced cell proliferation [20].

A recent study has evaluated the effects of metformin on PCa focusing on the AMPK pathway in paired pre-treatment and prostatectomy specimens [21]. Although the study was limited by small sample size and lack of a control arm, a change in the proliferation marker ki67 could be observed following metformin therapy (mean 50% reduction). Together with our collaborators at the Centre for Molecular Oncologic Pathology (CMOP), Dana Farber Cancer Institute (DFCI), we have also investigated the molecular pathways involved in PCa in a cohort of 181 men. Preliminary results from the Uppsala Longitudinal Study of Adult Men (ULSAM) cohort showed that men with higher levels of fatty acid synthase (FASN) had an increased risk of prostate cancer death compared to patients with normal levels (unpublished data). Furthermore, Flavin et al. have shown that lack of AMPK activity is associated with and may be an important biochemical alteration in MetS [22].

### Rationale for the study

A potential role for metformin in PCa has been suggested and given its wide availability, tolerable side effect profile and safety record it may represent a therapeutic option for men with PCa. However, the mechanism of action by which metformin exerts its anti-cancer effect has yet to be fully characterised. This ‘window of opportunity’ trial provides an opportunity to investigate this by comparing baseline prostate biopsies with post-treatment surgical specimen by focussing on assessment of the FASN/AMPK axis. This study will have a placebo arm in order to provide a control group. Non-diabetic patients with newly diagnosed PCa scheduled for radical prostatectomy will be eligible for treatment with metformin/placebo for four weeks prior to prostatectomy.

### Risk/benefit

Usual timing between diagnostic biopsy and prostatectomy is four weeks on average, so therefore it is not expected that surgery will be delayed as a result of participation in this study. Since this is a proof of principle trial with a relative short duration of treatment, it is unlikely that patients will derive significant benefit by study participation. However, it has been shown that metformin is well tolerated in a non-diabetic population [21, 23] and it is not anticipated that patients will experience increased morbidity by study participation.

### Trial design

This is a randomised, placebo-controlled, double-blind, window of opportunity study investigating the biological mechanism of metformin in PCa. Patients with newly-diagnosed, early stage, prostate cancer scheduled for radical prostatectomy will either enter the main study and be randomised 1:1 to receive metformin (2 g daily over 2 divided doses; Arm A) or placebo four weeks prior to prostatectomy (standard of care; Arm B). A subset of five patients will enter the exploratory PET-MRI sub study. These five patients will all receive metformin and will undergo an additional two PET-MRI Scans (see below).

Patients with a history of a current or historical diagnosis of diabetes mellitus and/or prior metformin use will be excluded.

The primary objective of this study is to investigate the biological mechanism of metformin on PCa using pharmacodynamic markers (Table 1). The primary endpoint for this study is the difference in expression levels of biomarkers representing the FASN/AMPK pathway for the metformin and placebo groups, as measured by the H score. Secondary endpoints include the difference in indicators of proliferation in the same groups, as well as differences in expression levels of the biomarkers between benign and malignant tissue (Table 1).

**Table 1:** Objectives

Objectives	Endpoints
Primary endpoints	
To determine the biological effect of metformin on markers of the FASN/AMPK pathway in prostate tissue by comparison of pre and post-treatment samples.	Assessment of the difference in expression levels of markers of the FASN/AMPK pathway pre and post treatment between the placebo and metformin arms.
Secondary endpoints	
To evaluate the biological effect of metformin on markers of proliferation in prostate tissue by comparison of pre and post-treatment samples.	Assessment of the difference in expression levels of indicators of proliferation (ki67 and Terminal deoxynucleotidyl transferase dUTP nick end labelling (TUNEL)) pre and post treatment between the placebo and metformin arms.
To evaluate differences in FASN/AMPK-associated markers in benign and malignant prostate tissue.	Assessment of the difference in expression levels of markers of the FASN/AMPK pathway and indicators of proliferation between benign and malignant prostate tissue in the placebo and metformin arms.
To measure metformin levels in prostate tissue.	Assessment of the difference in metformin levels in baseline and post-treatment prostate tissue.
To determine safety of metformin in this non-diabetic patient cohort.	Assessment of adverse events and laboratory evaluations.
To determine surgical toxicity.	Assessment of surgical-specific toxicities: time between biopsy and surgery, peri-operative bleeding, infection, rectal injury and length of hospital stay.
Exploratory Objectives and Endpoints	
To evaluate the effects of metformin on functional imaging of the prostate.	Difference in ^18^F Choline PET/MRI between baseline and post-treatment (prior to prostatectomy) in a separate non-randomised cohort of five patients with MRI positive disease receiving metformin.

Following informed consent (see Additional file 1: Appendix 1 for informed consent form) and screening, patients in the main study will be randomised and continue metformin or placebo for four weeks until the evening prior to radical prostatectomy. The five patients in the PET-MRI sub study will all receive metformin. In the event that surgery is scheduled for after this time point, patient will continue study drug for an additional one week. Prostate tissue (at baseline from biopsy and post treatment from prostatectomy) will be used for analysis of p-AMPK, p-ACC, FASN by immunohistochemistry and proliferation will be measured using ki67 and TUNEL in both groups.

Tissue metformin levels will also be assessed in baseline and post-treatment prostate tissue in the metformin-cohort. Each tissue specimen will be assessed by an experienced uro-pathologist to identify benign and malignant tissue. Patients will also be invited to consent for tissue storage in an HTA licensed Biobank. Additional translational studies may be undertaken based upon the results of the initial analysis as described in this protocol. Study drug safety will be assessed by recording adverse events.

The primary endpoint of this study is pharmacodynamic and therefore time between study drug dose and prostatectomy is an important factor. To minimise the effects of dose reductions and interruptions, the primary endpoint analysis will be based on a per protocol analysis. Evaluable patients are defined as:

-Received at least 21 days (3 weeks) of study drug between 1.5–2.0 g daily.

-Received study drug uninterrupted for the last 7 days prior to prostatectomy.

-A secondary analysis will include an intention-to-treat analysis.

Histopathological staging from prostatectomy will be performed. Following prostatectomy, all patients will be followed up for a final safety assessment and recording of surgical toxicity by the Clavien-Dindo system. Following this visit, patients do not require further study-related follow up and will continue to receive standard of care.

The exploratory endpoint of this study involves 18F Choline PET/MRI evaluation at baseline and post-metformin (pre-prostatectomy) for assessment of response in prostate tissue. This exploratory sub-study will include 5 patients with MRI positive disease, not randomised in the main trial, all of whom will receive metformin. Apart from the additional two visits for the 18F Choline PET/MRI scans they will follow the same trial protocol/visit schedule as those in the main study. The criteria for enrolment in to this sub study are:

1. Patient willing to undergo two additional PET-MRI scans
2. MRI positive disease
3. Satisfactory completion of MRI safety questionnaire
4. Availability of 18F Choline and scanning slots which would not result in a delay to the patient’s enrolment into the study or to their surgery

## Methods: Participants, interventions, and outcomes

### Study setting

The trial is currently open at two tertiary referral hospitals in London, UK.

- Guy’s and St Thomas NHS Foundation Trust
- Royal Marsden NHS Foundation Trust

Full details can be found on the EudraCT website https://www.clinicaltrialsregister.eu/ctr-search/search?query=2014-005193-11.

### Eligibility criteria

#### Inclusion criteria

Patients eligible to participate in this study are those who meet all of the following inclusion criteria:

1. Age 18 or older and willing and able to provide signed informed consent.
2. Histologically confirmed adenocarcinoma of the prostate, with a maximal tumour length of greater or equal to 6 mm on core biopsy
3. No previous treatment for prostate cancer (including surgery, any hormone therapy, radiotherapy and cryotherapy)
4. Prostate biopsy within 6 months from screening
5. Radical prostatectomy is the scheduled treatment of choice
6. Eastern Cooperative Oncology Group (ECOG) Performance status less than or equal to 0 or 1
7. Adequate organ function, defined as follows:
  Haemoglobin >10.0g/dL
  Absolute neutrophil count >1.5x10^9^/L
  Platelet count >100x10^9^/L
  Renal function, eGFR >60ml/min (calculated by Cockcroft Gault)
  AST and/or ALT <2.5 x ULN
  Total Bilirubin <1.5 x ULN
8. Able to swallow the drug and comply with study requirements.

#### Exclusion criteria

Patients must NOT meet any of the following exclusion criteria:

1. Patients with a current or historical diagnosis of type one or two Diabetes and/or have ever received metformin
2. Patients with hypersensitivity to any of the components of Metformin or placebo tablet
3. History of or conditions associated with lactic acidosis such as shock or pulmonary insufficiency, alcoholism (acute or chronic), and conditions associated with hypoxaemia
4. Patients with chronic liver disease, severe cardiovascular impairment, cardiac failure, recent myocardial infarction, severe peripheral vascular disease or renal impairment (eGFR <60 ml/min as measured by Cockcroft Gault)
5. Patients with acute severe disorders, for example infections with fever, pancreatitis, trauma, dehydration or reduced diet (<1000 kcal or 4200 kJ per day)
6. Other active malignancy over the last five years that has required systemic therapy, excluding:
  - Adjuvant therapy in the curative setting
  - Non-melanoma skin cancer
  - Superficial transitional cell carcinoma (CIS-T1)
7. Current enrolment in an investigational drug or device study or participation in such a study within 30 days of signing consent.
8. Any subjects who is able to father a child and does not agree to use barrier protection, in the form of a condom, for the duration of the trial and for 16 weeks after the last study drug administration.

### Interventions

Screening procedures within 14 days of consent

- Written informed consent from all participants
- Clinical assessments:
  - Complete medical history, including diagnosis, history of other diseases (active or resolved), concomitant illnesses and medications.
  - Record of patient demographics
  - Physical examination including vital signs, height and weight, waist/hip ratio and ECOG performance status
- Laboratory determinations: Blood results taken within 14 days of consent for other purposes can be used as part of the screening process:Full Blood Count (FBC), renal function, liver Function Tests (LFT), bone profile, fasting glucose, PSA, testosterone, fasting lipid profile, HbA1c. Select sites will also take two additional samples for a whole blood and serum save. This will be taken according to trial specific SOP (see Additional file 2: Appendix 2).
- Radiological assessment:
  - MRI Safety Assessment
  - In subgroup of 5 patients with MRI positive disease receiving metformin: ^18^ F Choline PET/MRI
- Tissue collection: Formalin Fixed Paraffin embedded tissue will be collected from baseline diagnostic specimen.

Study week 1 (day 1):

- Clinical assessments:
  - Physical examination including ECOG performance status if greater than 7 days from screening physical examination
  - Baseline adverse events
  - Medication review
  - Given compliance diary

Study week 3 (+/− 2 days):

- Clinical assessments:
  - Physical examination, including ECOG performance status and vital signs.
  - Adverse events
  - Compliance evaluation (diary and verbal)
  - Medication revieW

- Laboratory determinations: Blood tests taken within 2 days of compliance visit for other purposes can be used as part of the compliance visit
  - FBC, renal function, LFT, bone profile

Study week 4 (+/− 1 week) pre-prostatectomy:

- Clinical assessments:
  - Physical examination, including ECOG performance status, weight, waist/hip ratio and vital signs
  - Adverse events
  - Compliance evaluation
  - Medications review
- Laboratory determinations: Blood tests taken within 2 days of surgery visit for other purposes can be used as part of the surgery visit: FBC, Renal function, LFT, bone profile, fasting glucose, PSA, testosterone, fasting lipid profile. Select sites will also take two additional samples for a whole blood and serum save. This will be taken according to trial specific SOP (see Additional file 2: Appendix 2).

- Radiological assessment: In a subgroup of 5 patients with MRI positive disease: ^18^ F Choline PET/MRI, which will be performed after 21+/− 2 days of metformin and prior to prostatectomy. A time point prior to 28 days is chosen to allow radiological assessment to be scheduled without interfering with surgery scheduling.

- A pre-operative surgical visit should occur prior to surgery, as per standard of care and local policies.

Study week 4 (+/− 1 week) – prostatectomy:

Patients will undergo prostatectomy. This will occur at the end of week 4 (+/− 1 week). Study drug treatment will continue up until the evening before surgery until the patient is nil by mouth (as per local guidelines). In the events patients undergo surgery beyond 4 weeks from randomisation; study drug will be continued for an additional 1 week. Surgery should occur as per local policies.

- If clinically necessary, surgery can be brought forward or not performed (this should be discussed with the Chief Investigator). The case should be presented to a multidisciplinary team meeting before any other non-surgical treatment is given. These patients will not be included in the analysis as, in the absence of surgery, it will not be possible to assess for post-treatment tissue markers.

- Tissue Collection: Formalin Fixed Paraffin Embedded tissue will be taken from the radical prostatectomy specimen

Follow up 8–10 weeks post operatively

- Clinical assessments:
  - Symptoms directed physical examination, including ECOG performance status, weight, waist/hip ratio and vital signs
  - Medications review
  - Adverse events and complete Clavien Dindo assessment
- Laboratory determinations: Blood tests taken within 2 days of post-operative visit for other purposes can be used as part of the post-operative visit: FBC, renal function, LFT, bone profile, PSA, testosterone.

### Laboratory tests

Laboratory determinations including FBC, Renal function, LFT, bone profile, fasting glucose, PSA, testosterone, fasting lipid profile and HbA1c will be carried out by the local haematology and biochemistry laboratory at each site in accordance with local procedures.

Formalin fixed paraffin embedded tissue will be collected from baseline diagnostic biopsy and from the prostatectomy. Tissue will then be shipped to CMOP at DFCI. Samples will be processed and stored as per Laboratory Standard Operating Procedures.

The following analyses will be conducted at the CMOP on collected baseline and post-surgery tissue specimens:

- p-AMPK, p-ACC, FASN, ki-67 and TUNEL will be assessed in benign and malignant tissue by immunohistochemistry using image analysis.
- The ki-67 proliferation index is assessed by point counting 1000 cells, and is reported as percent positive cells.
- TUNEL is an apoptotic index defined as the number of apoptotic cells per 1000 tumour cells.
- Remaining markers will be measured using a H-score.

Methods for these analyses have been optimized and used in preliminary studies performed in collaboration at CMOP. Tissue (prostate) metformin concentrations will also be performed.

### Radiological assessment

During screening all five men undergoing 18F Choline PET/MRI will have successfully completed a MRI standard safety questionnaire (including eGFR) and their diagnostic clinical MRI will be have been checked to ensure it has visible disease. The patient will be asked to be nil by mouth 4 h prior the the scan. The scans will consist of:

- MRI Sequences: Prostate T1 and T2-weighted images
- prostate diffusion-weighted images
- BOLD MRI and MR spectroscopy.

Dynamic contrast enhanced MRI of prostate (0.1 mmol/kg IV). PET acquisition: 350 MBq 18F–choline IV. Dynamic image acquisition over pelvis. Patients will receive IV buscopan and undergo rectal filling as per standard MRI operating procedures.

### Dosing regimen

In order to limit gastrointestinal side effects patients will be instructed to take study drug at doses increasing from:

- 500 mg once a day (day 1–2)
- 500 mg twice a day (day 3–4)
- 1 g twice a day from day 5 onwards for 4 weeks until prostatectomy +/− one week

Study drug will be continued until the evening prior to surgery. Placebo will be dose escalated in the same way. Participants will be given these instructions verbally as well as written instructions at the start of their medication compliance diary.

Study drug doses should ideally be taken at the same time each day. Missed doses of the study drug may be taken later, provided that the time of dosing is at least 6 h before the next scheduled dose. If dosing is missed for one day for any reason, double-dosing should not occur the following day. Acute alcohol intoxication can increase the likelihood of the rare, but serious adverse event of lactic acidosis. Therefore, all participants will be advised to avoid alcohol for the duration of the trial. Patients participating in the non-randomised 18F PET/MRI cohort will receive metformin, which will be dose escalated as outlined above. All dose modifications and duration of treatment will be identical to the randomized cohort.

### Dose reduction in case of adverse events

The investigator should determine if an adverse event is related to the study drug. Adverse events (AE) considered at least possibly related to study drug may require a dose reduction, a temporary hold (up to 7 days), or permanent discontinuation. Dose modifications should be based on the NCI CTCAE (version 4). Dose reduction for Grade 1 AEs is not required. Dose reduction for Grade 2 events should be considered only when the AE is judged by the investigator to be clinically intolerable. For Grade 3 and 4 AEs, the dose modification of study drug should follow the Dose Reduction Guidelines in the Tables 2 and 3 below. Dose modification for Grade 3 or 4 diarrhoea should follow the guidelines in Table 4 below.

**Table 2:** General dose reduction guidelines

Grade I	Continue study treatment at same dose; monitor and treat as clinically indicated.
Grade II	Continue study treatment at same dose; monitor and treat as clinically indicated.
Grade III	Step 1. Interrupt study drug until toxicity reduced to ≤Grade 1. Step 2. Restart study treatment at same dose or lower dose at discretion of investigator
Grade IV	Step 1. Interrupt study drug until toxicity reduced to ≤Grade 2. Step 2. Restart study treatment at lower dose level.

**Table 3:** Dose level dose

Dose level	Dose
0	1 g BD.
-1	500 mg BD
-2	Discontinuation

**Table 4:** Dose reduction for specific toxicity: diarrhoea

Grade I	No action required.
Grade II	Concomitant anti-diarrhoeal agents may initially be administered without dose reduction. If Grade 2 diarrhoea persists, dose reduction should occur as per Table 2. Supportive care regimen should follow local standard of care.
Grade III	Dose reduction should occur as per Table 2.
Grade IV	Dose reduction should occur as per Table 2.

### IMP risks

As Metformin is a licensed drug the reference document will be the Medley Pharma laboratories Summary of Product Characteristics (SmPC). The very common unwanted effects (less than or equal to 1 in 10) are gastrointestinal symptoms such as nausea, vomiting, diarrhoea, abdominal pain and loss of appetite.

Lactic acidosis is a rare, but serious, metabolic complication that can occur due to metformin accumulation. Reported cases of lactic acidosis in patients on metformin have occurred primarily in diabetic patients with significant renal failure. The incidence of lactic acidosis can and should be reduced by assessing other associated risk factors such as poorly controlled diabetes, ketosis, prolonged fasting, excessive alcohol intake, hepatic insufficiency and any condition associated with hypoxia. For full details please refer to the SmPC.

### Drug accountability

The pharmacy will keep accountability records for reconciliation purposes. These should be used to record the identification of the subject to whom the investigational product was dispensed, the date, batch number, expiry date and quantity of the investigational product dispensed and the quantity of the investigational product unused/returned by the subject. Participants will be asked to return all packaging to pharmacy for accountability. Any excess or unused drug will be collected by the trial team, retained for verification by the local Clinical Research Associate (CRA) and destroyed by Guy’s Hospital Pharmacy in accordance with local requirements when authorised to do so. Disposal of unused investigational medicinal product (IMP) is only permitted with sponsor authorisation.

### Storage of IMP

This IMP does not require any special storage conditions. IMP should be handled and stored safely and properly in accordance with the drug label. Patients will be instructed to store study drug at room temperature out of reach of children.

### Subject compliance

Trial subjects will undergo a compliance evaluation at their Study week 3 (+/− 2 days) visit. This will consist of reviewing a medication diary given at enrolment and a verbal questioning about drug compliance.

### Concomitant medication

For management of concomitant therapies, please refer to the SMPC.

### Participant timeline

**Fig. 1:**
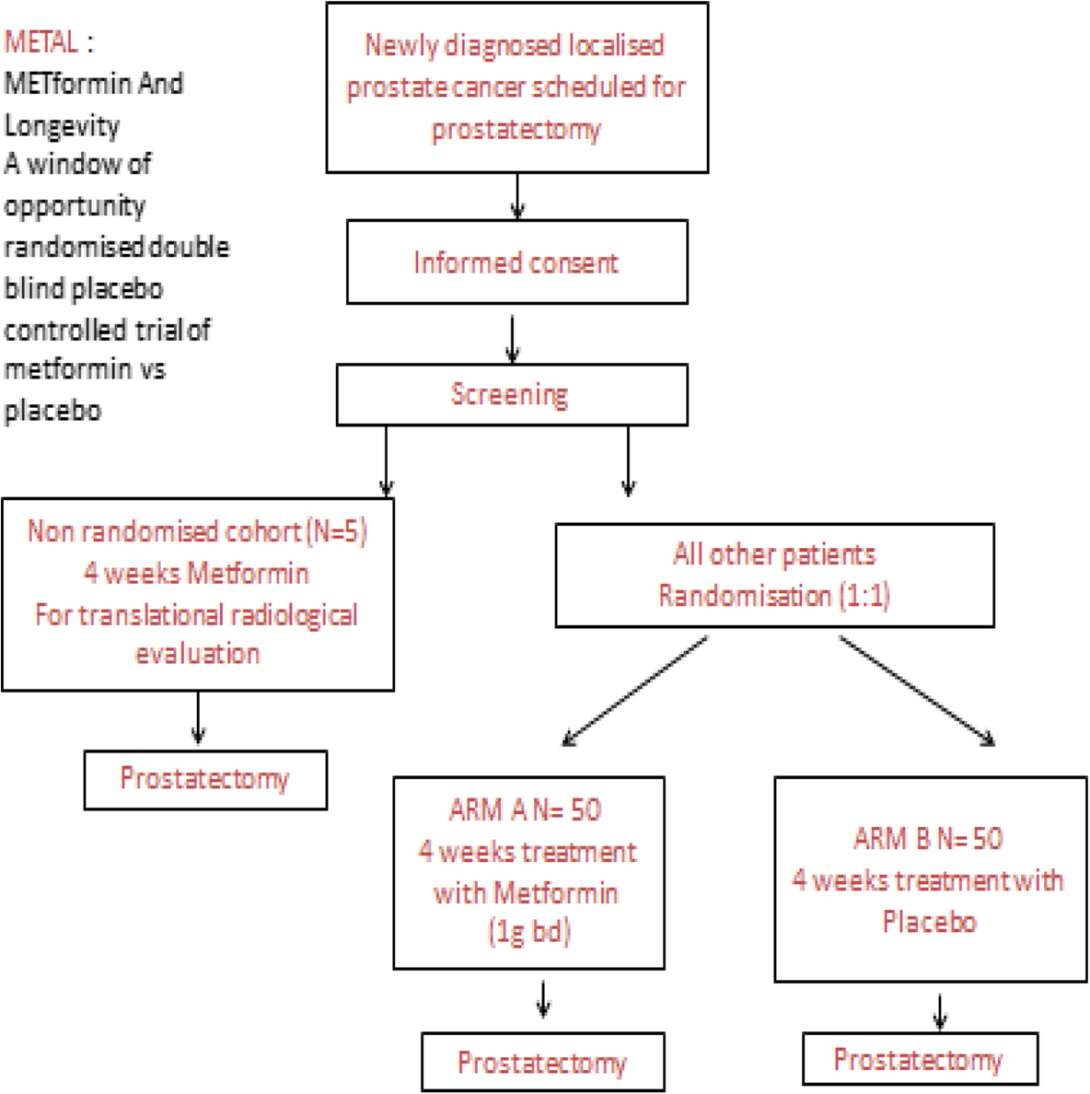
Trial Schema

Please see below Fig. 1 for the trial schema and Table 5 for the trial flow chart.

**Table 5:** Trial Flow Chart

Phase	Screening	Pre-surgery Treatment			Surgery	Post-surgery
Time point	≤14 days before baseline	Baseline Day 1 of treatment	Day 21 (+/- 2 days)	Day 28 (+/- 1 week) prior to surgery	Day 28 (+/- 1 week)	8-10^f^ weeks post-op
Informed consent	x					
Eligibility review	x	x				
Randomisation		x				
Medical History^a^	x					
Demographics	x					
Physical Exam	x	x	x	x		x
Vital signs^b^	x		x	x		x
ECOG PS	x	x	x	x		x
Height	x					
Weight	x			x		x
Waist/Hip ratio	x			x		x
Haematology	x		x	x		x
Biochemistry^c^	x		x	x		x
Fasting Glucose/Lipids	x			x		
PSA and Testosterone	x			x		x
HbA1c	x					
Whole blood and Serum save^d^	X			X		
Study Drug Administration		x	x	x		
Medication review	x	x	x	x		x
Compliance evaluation (diary and verbal)			x	x		
Adverse events (CTCAE v4)^e^		x	x	x		x
Paraffin embedded tissue sent to laboratory	x				x	
Prostatectomy					x	
MRI safety assessment^g^	x					
^18^F Choline PET/MRI^g^	x			X^e^		

^a^Full medical history, including history other disease, active or resolved, concomitant illnesses and cancer diagnosis

^b^Blood pressure, pulse rate and oxygen saturation, BM

^c^Renal profile, liver function tests, bone profile

^d^To be taken at selected sites only and according to the Trial specific SOP

^e^Clavien Dindo assessment to be completed at 8-10 weeks post operatively

^f^This review will coincide with routine post-operative review

^g^Only for the 5 subjects participating in the exploratory PET-MRI group

### Sample size

The primary analysis for this study will quantify the difference in expression levels of biomarkers representing the FASN/AMPK pathway, as well as indicators of proliferation, for the metformin and placebo groups as measured by the H score using a simple two-sample t-test. Secondary analyses will include a comparison of differences in expression levels of biomarkers of the FASN/AMPK pathway, as well as indicators of proliferation, between benign and malignant tissue. Finally, we will perform a multivariate regression analysis to predict effects of metformin on expression levels using tumour and patient-specific characteristics.

Our original sample size calculation was based on the H-score used to assess expression levels of the studied biomarkers, which ranges from 0 to 300. We conducted a two-sided test (alpha = 0.05; power = 0.80) comparing the mean difference in the two groups for different scenarios as we will be testing different biomarkers. Based on these scenarios, we planned to recruit 90 patients for each arm over a period of 15 months. However, since the start of our trial we have also identified other pathways to be studied in the prostate tissue. Moreover, we will set up a stratification trial following the biological information obtained in this trial. As a result we have reviewed our sample size calculation by increasing the type I error to 20% - which will require us to only recruit 50 men in each group. As we will conduct a follow-up trial with a clinical outcome, the potential type I error can be corrected for in this second trial. At the current stage it is thus more important to reduce the probability of failing to reject the null hypothesis when it is false. Hence, we have not changed the power in our revised sample size calculation. Table 6 below shows the revised power calculation. In addition to the 50 patients in each arm, we will recruit an additional five patients in the exploratory endpoint group who will not be randomised.

**Table 6:** Sample size calculation (two-sided test with power = 0.80) to identify mean difference in H score between biopsy and radical prostatectomy specimen for the metformin and control group

	Mean Difference (SD) in Metformin Group	Mean Difference (SD) in Control Group	N needed with α = 0.05	N needed with α = 0.10	N needed with α = 0.20
Scenario 1	15 (35)	0 (35)	86	38	50
Scenario 2	30 (65)	0 (65)	74	59	43
Scenario 3	20 (25)	5 (25)	44	35	26
Scenario 4	30 (50)	5 (50)	63	50	37

### Recruitment

Patients will be identified in multi-disciplinary team meetings or in out-patient clinics by the clinical team. Only patients with adequate diagnostic prostate biopsy specimen available for baseline immunohistochemistry will be approached for participation in this study. Assessment of this will be undertaken by an experienced uro-pathologist present during multi-disciplinary team meetings. Patients will be selected to be approached for recruitment in to either the sub study or the main study, depending on whether the criteria for the sub study are fulfilled. Patients approached about the sub study, will be able to opt for enrolment in to the main study should they wish. Once all five patients are recruited to the sub study, all subsequent patients will be approached only about the main study.

## Methods: Assignment of interventions

### Randomisation

Patients will be randomised using block randomisation with randomly varying block sizes. Randomisation will be performed via a web based independent randomisation service, hosted at the UKCRC registered KCTU. Researchers will access the system via http://www.ctu.co.uk and will login with individual usernames and passwords. When a patient is confirmed as eligible and consenting, their study ID, initials, and date of birth will be entered into the system, along with any relevant stratification information, and the patient will be randomised to active or placebo medication. The system will auto-generate confirmation emails to pharmacy advising of the trial arm to be dispensed. A blinded confirmation email will be generated to the rest of the research team.

### Emergency code break

Investigators and patients will remain blinded to the treatment allocation throughout the trial. Unblinding should not normally be necessary as serious side-effects should be dealt with on the assumption that the patient is on active metformin treatment. Study medication should be omitted rather than unblinded. Request for unblinding should be directed to local pharmacy during office hours. In case of emergency un-blinding being necessary out of hours, the on call pharmacist should be contacted. Contact details for individual sites will be provided on site specific emergency contact list.

### Withdrawal of patients

Participants have the right to withdraw from the study at any time for any reason. The investigator also has the right to withdraw patients from the study drug in the event of inter-current illness, AEs, SAE’s, SUSAR’s, protocol violations, administrative reasons or other reasons. It is understood by all concerned that an excessive rate of withdrawals can render the study un-interpretable; therefore, unnecessary withdrawal of patients should be avoided. Should a patient decide to withdraw from the study, all efforts will be made to report the reason for withdrawal as thoroughly as possible. Should a patient withdraw from study drug only, efforts will be made to continue to obtain follow-up data, with the permission of the patient.

Participants who wish to withdraw from IMP will be asked to confirm whether they are still willing to provide the following.

- trial specific data at their follow up visit

Patients who interrupt study drug for greater than 7 days, without the direction from their treating doctors, will be considered as non-compliant and will be discontinued from the study. These patients will be included in the safety assessments. They will be included in the pharmacodynamic, efficacy and safety assessments only if they received at least 21 days of treatment.

Treatment with study drug should be discontinued if it is considered to be in the best interest of the patient. Reasons for treatment discontinuation include:

- Disease progression
- Occurrence of intolerable side effects
- Patient withdrawal of consent or non-compliance.

Patients discontinued from the study for reasons unrelated to therapy, such as non-compliance, ineligibility or withdrawal of consent will be considered drop-outs. All of these patients are still evaluable for toxicity. Any subjects who withdraw prior to completing treatment will be replaced until 90 subjects in each of the randomized study arms have completed treatment.

## Methods: Data collection, management and analysis

A separate data management plan will be created for the trial. The case report forms will be paper based. They will be collated and completed by the clinical trial co-ordinator and dedicated research nurse. A password protected database will be created on the ACCESS platform to allow speed of data entry.

The Chief Investigator will act as custodian for the trial data. The following guidelines will be strictly adhered to: Patient data will be anonymised*.*

- All anonymised data will be stored on a password protected encrypted computer.
- All trial data will be stored in line with the Medicines for Human Use (Clinical Trials) Amended Regulations 2006 and the Data Protection Act and archived in line with the Medicines for Human Use (Clinical Trials) Amended Regulations 2006 as defined in the Kings Health Partners Clinical Trials Office Archiving SOP.

### Per protocol analysis

The primary endpoint of this study is pharmacodynamic and therefore time between study drug dose and prostatectomy is an important factor for evaluation of the primary endpoint. To minimize the effects of dose reductions and interruptions, the primary endpoint analysis will be based on a per protocol analysis. Evaluable patients are defined as:

- Received at least 21 days (3 weeks) of study drug between 1.5–2.0 g daily.
- Received study drug uninterrupted for the last 7 days prior to prostatectomy.

### Intention-to-treat population

The intention-to-Treat (ITT) population is defined as all patients who were randomised in this study. The ITT population will be analysed by treatment arm as randomised (i.e. treatment arm based on randomisation assignment).

### Safety analysis

The safety population is defined as all randomised patients who received at least 1 dose or partial dose of study drug. The safety population will be analysed by treatment arm as treated. The safety population will be used to conduct safety analyses.

### Exploratory analysis

Exploratory analysis by ^18^F Choline PET/MRI will be performed in five patients with MRI positive disease who will not be randomised and will all receive metformin. This patient population will be used to conduct exploratory analyses. Once five complete datasets are completed no further recruitment to this group will occur. Data will be summarised descriptively.

### Accrual and duration of study

The estimated accrual for this study is 10 patients a month. Allowing for a 5% drop out rate, patient accrual is expected to be completed within 18 months. We will account for all of the patients registered in the study. The number of patients who were not evaluable, who died or withdrew before treatment began will be specified. The distribution of follow-up time will be described and the number of patients lost to follow-up will be given.

## Methods: Monitoring

Neither the co-sponsors nor the investigators felt this study warranted a data monitoring committee (DMC) given the use of metformin which is a safe, well tolerated post licensed drug.

### Reporting responsibilities

Organisations have delegated the delivery of the Sponsor’s responsibility for Pharmacovigilance (as defined in Regulation 5 of the Medicines for Human Use (Clinical Trials) Regulations 2004 to the King’s Health Partners Clinical Trials Office (KHP-CTO).

All SAEs, SARs and SUSARs will be reported immediately by the Chief Investigator (and certainly no later than 24 h) to the KHP-CTO in accordance with the current Pharmacovigilance Policy. Death as a result of disease progression and other events that are primary or secondary outcome measures are not considered to be SAEs and should be reported in the normal way, on the appropriate CRF.

The KHP-CTO will report SUSARs to the regulatory authorities Medicines and Healthcare products Regulatory Agency (MHRA), competent authorities of other European Economic Area states in which the trial is taking place.

The Chief Investigator will report to the relevant ethics committee. Reporting timelines are as follows:

- SUSARs which are fatal or life-threatening must be reported not later than 7 days after the sponsor is first aware of the reaction. Any additional relevant information must be reported within a further 8 days.
- SUSARs that are not fatal or life-threatening must be reported within 15 days of the sponsor first becoming aware of the reaction.

The Chief Investigator and KHP-CTO (on behalf of the *co*-sponsors) will submit a Development Safety Update Report (DSUR) relating to this trial IMP to the MHRA and REC annually. Monitoring of this trial will be to ensure compliance with Good Clinical Practice and scientific integrity will be managed and oversight retained, by the KHP-CTO Quality Team.

### Ethics and dissemination

The trial will be conducted in compliance with the principles of the Declaration of Helsinki (1996), the principles of GCP and in accordance with all applicable regulatory requirements including but not limited to the Research Governance Framework and the Medicines for Human Use (Clinical Trial) Regulations 2004, as amended in 2006 and any subsequent amendments.

This protocol and related documents have been submitted for review to Fulham Research Ethics Committee (REC), and to the MHRA for Clinical Trial Authorisation, as will all substantial and non-substantial amendments. The Chief Investigator will submit a final report at conclusion of the trial to the KHP-CTO (on behalf of the Sponsor), the REC and the MHRA within the timelines defined in the Regulations.

The co-sponsors, King’s College London and Guy’s and St Thomas NHS Foundation Trust, will provide insurance and indemnity. Funding to conduct the trial is provided kindly by the JP Moulton Charitable Foundation. It is intended that the results of the study will be reported and disseminated at international conferences and in peer-reviewed scientific journals.

## Discussion

This multi-site randomised placebo-controlled double blinded trial of metformin vs. placebo in men with localised prostate cancer due to undergo radical prostatectomy aims to elucidate the mechanism of action of metformin in PCa cells, which should then enable further larger stratification trials using metformin in different stages of PCa to take place.

## Additional files

Additional file 1: Appendix 1. Informed Consent Form. (DOC 213 kb)

Additional file 2: Appendix 2. SOP serum and whole blood preperation. (DOCX 23.8 kb)

## Abbreviations

AE: Adverse event
AMPK: Activated protein kinase
CMOP: Centre for Molecular Oncologic Pathology (CMOP)
CRA: Clinical research associate
DFCI: Dana Farber Cancer Institute (DFCI)
DMC: Data monitoring committee
FASN: Fatty acid synthase
FBC: Full blood count
IGF-1: Insulin like growth factor − 1
IMP: Investigational medicinal product
ITT: Intention to treat
KCTU: King’s College clinical trial unit
LFTs: Liver function tests
METAL: Metformin and longevity
MetS: Metabolic syndrome
MHRA: Medicines and Healthcare products Regulatory Agency
MTOR: Mammalian target of rapamycin
NCI CTCAE: National cancer institute common terminology criteria for adverse events
PCa: Prostate cancer
PSA: Prostate specific antigen
REC: Research ethics committee
SAE: Serious adverse event
SAR: Serious adverse reaction
SMPC: Summary of product characteristics
SOP: Standard operating procedure
SUSAR: Suspected unexpected serious Adverse Reaction
T2DM: Type two diabetes mellitus
TUNEL: Terminal deoxynucleotidyl transferase dUTP nick end labelling
ULSAM: Uppsala Longitudinal Study of Adult Men
