# Supplementary material for: Metformin and longevity (METAL): a window of opportunity study investigating the biological effects of metformin in localised prostate cancer
Source: BMC Cancer. 2017 Jul 21;17:494. doi: 10.1186/s12885-017-3458-3 (PMC5520293; doi:10.1186/s12885-017-3458-3)
Supplement: Supplementary file 1 — Informed Consent Form. (DOC 213 kb) [file 12885_2017_3458_MOESM1_ESM.doc]

**
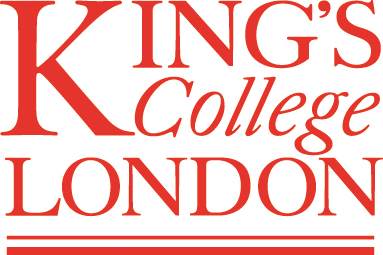
**

#
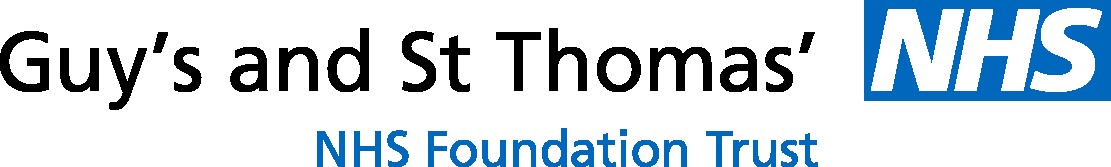


# MAIN STUDY - CONSENT FORM

**METAL: METformin And Longevity**

A window of opportunity study investigating the biological effects of metformin in localised prostate cancer

**Participant Identification Number: ………………………………………**

**Name of U.K. Institution leading the Research : Guy’s & St Thomas’ NHS Trust**

**Research Director: Dr Sarah Rudman**

**Phone Number and Contact Details: Fee Cahill**

**Prostate Research Nurse**

**07464492509Bleep 2155**

| **Please initial box to indicate agreement** | | | | | | | |
| --- | --- | --- | --- | --- | --- | --- | --- |
|  | | | | | | | |
|  | | | | | | | |
| 1 | I confirm that I have read and understand the patient information sheet (Version ....... dated ............................) for the above study. I have had the opportunity to consider the information, ask questions and have had these answered satisfactorily. | | | | |  |  |
| 2 | I understand that my participation is voluntary and that I am free to withdraw at any time, without giving any reason, without the standard of my medical care or legal rights being affected. | | | | |  |  |
| 3 | I understand that relevant sections of any of my medical notes and data collected during the study may be looked at by responsible individuals from regulatory authorities or from the Guy’s and St Thomas NHS Foundation Trust / King’s College London, where it is relevant to my taking part in this research. I give permission for these individuals to have access to my records. I understand that this will mean my data may be sent outside of the UK and EEA. | | | | |  |  |
| 4 | I agree to the donation of blood and tissue samples to be given at the timelines specified in the above mentioned information sheet. I agree that samples can be stored at the King’s Health Partners Cancer Biobank, Guy’s Hospital, London for current and future research. I understand that this research may involve laboratories outside of the UK and EEA. | | | | |  |  |
| 5 | I agree to the donation of tissue samples for future genetic research. This potentially includes freezing and storage of tissue for future research. Blood cells will not be used for reproductive cloning and inherited disease research. I understand that this research may involve laboratories outside of the UK and EEA. | | | | |  |  |
| 6 | I agree to my GP being informed of my participation in the study. | | | | |  |  |
| 7  8 | I agree to to use a method of birth control with adequate barrier protection as determined to be acceptable by the chief investigator during the study and for 16 weeks after the last study drug administration  I agree to take part in the above study | | | | |  |  |
|  |  | | | | |  |  |
|  | |  |  |  |  | |  |
| Name of Patient | |  | Date |  | Signature | |  |
|  | |  |  |  |  | |  |
| Name of Person taking consent | |  | Date |  | Signature | |  |
|  | |  |  |  |  | |  |

**3 copies: 1 for patient, 1 for research team, 1 to be kept with hospital notes**
