# Supplementary material for: Metformin and longevity (METAL): a window of opportunity study investigating the biological effects of metformin in localised prostate cancer
Source: BMC Cancer. 2017 Jul 21;17:494. doi: 10.1186/s12885-017-3458-3 (PMC5520293; doi:10.1186/s12885-017-3458-3)
Supplement: Supplementary file 2 — SOP serum and whole blood preperation. (DOCX 23.8 kb) [file 12885_2017_3458_MOESM2_ESM.docx]

**METformin And Longevity (METAL): A window of opportunity study investigating biological effects of metformin in localised prostate cancer**

EudraCT number 2014-005193-11

IRAS number: 151495

**SOP Serum preparation for METAL trial samples**

------------------------------------------------------------------------------------------------

1. First let 4.5 mls of blood in red-top vacutainer clot for 30 min and process within 2 hours of the draw
2. Centrifuge vacutainer tubes for 13 min at 4000rmp at room temperature
3. Collect 1ml aliquots of serum from the top layer in cryovials
4. Store cryovials in -80C freezer

**SOP whole blood preparation for METAL trial samples**

------------------------------------------------------------------------------------------------

1. Then let 4 mls of blood in to an EDTA tube
2. Invert the tube 5-6 times
3. EDTA samples can be kept at room temperature
4. Remove 3 ml of blood from the EDTA tube and aliquot 1 ml into 3 cryovial tubes which are pre-labelled with patients METAL trial ID and marked WB (whole blood)
5. Freeze samples within 1 hour of blood draw, store cryovials in a designated -80◦C freezer
